# Co-Creation and Usability Testing of a Digital Cardiac Rehabilitation Program (eCardiacRehab): Design and Development Study

**DOI:** 10.2196/93398

**Published:** 2026-09-01

**Authors:** Oda Karin Nordfonn, Kristin Johnsen Ramstad, Trond Roed Pettersen, Nina Hjertvikrem, Inger-Lise Aamot Aksetøy, Alf Johannes Agcaoili Borge, Eva Gerdts, Jan Gunnar Hesthammer, Torstein Hole, Irene Instenes, Anita Isaksen, Ole Andreas Krumsvik, Merethe Landaas, Christian Moltu, Svein Rotevatn, Jan Schjøtt, Cathrine Horn Sommersten, Jan Ove Tryti, Tone Merete Norekvål

**Affiliations:** 1 Department of Heart Disease, Haukeland University Hospital, Jonas Lies Vei 65, Bergen, Vestland, 5021, Norway, 47 53491472; 2 Department of Health and Caring Sciences, Western Norway University of Applied Sciences, Stord, Vestland, Norway; 3 Department of Health and Caring Sciences, Faculty of Health and Social Sciences, Western Norway University of Applied Sciences, Bergen, Vestland, Norway; 4 National Advisory Unit on Exercise Training as Medicine for Cardiopulmonary Conditions, St Olav's University Hospital, Trondheim, Trøndelag, Norway; 5 Department of Circulation and Medical Imaging, Norwegian University of Science and Technology, Trondheim, Trøndelag, Norway; 6 Landsforeningen for hjerte, lunge og hjerneslag, LHL, Bergen, Vestland, Norway; 7 Department of Clinical Science, University of Bergen, Bergen, Vestland, Norway; 8 Section for eHealth, Haukeland University Hospital, Bergen, Vestland, Norway; 9 Medical Department, Helse Møre og Romsdal HF, Ålesund, Møre og Romsdal, Norway; 10 Department of Public Health end Nursing, Norwegian University of Science and Technology, Trondheim, Trøndelag, Norway; 11 Youwell AS, Bergen, Vestland, Norway; 12 Department of Health and Caring Sciences, Western Norway University of Applied Sciences, Førde, Vestland, Norway; 13 Department of Psychiatry, Helse Førde, Førde, Vestland, Norway; 14 Department of Ageing, Health and Care, Kunnskapskommunen Helse Omsorg Vest, Bergen Kommune, Bergen, Vestland, Norway; 15 Sogndal Municipality, Sogndal, Vestland, Norway

**Keywords:** co-creation, co-design, digital cardiac rehabilitation, multidisciplinary, user experience, eHealth

## Abstract

**Background:**

Cardiac rehabilitation (CR) is a class IA–recommended treatment and secondary preventive strategy for people with coronary artery disease. eHealth solutions show promise in providing accessible CR. However, few provide a comprehensive and multimodal CR program, and most programs are developed without input from end users.

**Objective:**

The aim of this study was to provide a detailed description of the multidisciplinary co-creation process used to develop a person-centered, multimodal digital CR program that integrated the perspectives of stakeholders and end users throughout, with the rationale to optimize the acceptance and feasibility of the CR program.

**Methods:**

We incorporated a user-centered design with a 3-phase, iterative, multidisciplinary stakeholder co-creation process. This included *defining needs* (phase 1), *development and refinement* (phase 2), and *testing and optimization* (phase 3). Overall, 26 internal stakeholders from the project team participated, including clinicians, researchers, software developers, and user representatives. In addition, 35 external stakeholders (20 clinicians and 15 people with cardiac-related problems) participated in a user-centered development process that included workshops, intervention content development, and usability testing. Data were collected through contextual inquiry, 4 workshops with stakeholders, 12 co-creation sessions with user representatives, 2 usability testing sessions using a think-aloud methodology, standardized usability surveys, and individual and focus group interviews. Intervention content was developed and finalized based on existing evidence, stakeholder input, and user testing. A smoke test and a minimum viable product test were conducted. Data were analyzed using rapid qualitative content analysis and descriptive statistics.

**Results:**

Analyses of data gathered across the 3 phases identified 5 themes important to stakeholders and end users: a program with (1) personalized and tailored solutions with reliable, trustworthy, and evidence-based content (ie, a face-to-face introduction session at the start of the program and a tailored registration page to individually register personal risk factors, goals, and medications); (2) options for feedback (ie, an asynchronous messaging service and tailored face-to-face video consultations with clinicians); (3) peer support (ie, exercise sessions in groups); (4) digital reminders (ie, notifications on a smartphone or tablet); and (5) motivational features (ie, interactive cardiac-specific learning modules on coronary artery disease and secondary prevention). Evidence-based content development resulted in 9 cardiac-specific interactive learning modules, a module for personal health data entry, live exercise sessions, tailored digital follow-up, and an asynchronous messaging service. Readability was assessed using the Gunning Fog Index, with levels ranging from 4 to 13. The overall System Usability Scale score was 86.2 (SD 12.2), indicating excellent usability.

**Conclusions:**

A multidisciplinary stakeholder co-creation process combining well-known evidence-based concepts with stakeholder input identified several areas for improvement and facilitated the development of the digital CR program. Contextually tailored, co-designed interventions may improve relevance and responsiveness to user needs. The feasibility and effectiveness of the program will be further evaluated in the eCardiacRehab trial.

## Introduction

Coronary artery disease (CAD) remains a significant global health burden, accounting for substantial morbidity and mortality despite advances in acute care and secondary prevention strategies [1]. Cardiac rehabilitation (CR) is a well-established, evidence-based secondary prevention intervention shown to reduce mortality, improve functional capacity, and enhance quality of life among people with CAD [2]. Accordingly, CR is a class 1A–recommended intervention in European guidelines [3]. Despite these recommendations, only 55% of countries worldwide offer CR [4]. Although the benefits of CR are well documented as a clinically effective model of care, participation rates range from 14% to 35% [5]. Reasons for low participation in CR include limited access, low referral rates, and low adherence [5]. In Norway alone, more than 11,000 patients undergo percutaneous coronary intervention (PCI) annually, which is an indication for CR. Nevertheless, the national average CR participation rate is only 14% [6].

The increasing integration of eHealth technologies into cardiac care offers new opportunities to address challenges in CR by enabling remote, flexible, and scalable delivery of health care services [7]. Digital CR programs hold promise for improving access and adherence; however, their effectiveness depends critically on user engagement, acceptability, and the alignment of intervention design with user needs and clinical workflows [8,9]. User engagement significantly influences the acceptability of programs. However, optimizing user contributions during program development poses challenges [10].

Co-creation has emerged as a pivotal approach in the development of eHealth interventions, emphasizing the active collaboration of multidisciplinary and diverse stakeholders—patients, clinicians, designers, and researchers—at all stages of the design process [11]. This participatory methodology fosters a deeper understanding of user needs, preferences, and contextual factors, which are essential for creating interventions that are not only clinically effective but also acceptable, feasible, engaging, and motivating for end users [12]. By promoting shared ownership and mutual learning, co-creation enhances the relevance and sustainability of eHealth solutions, ultimately improving implementation, patient adherence, and health outcomes [13]. This methodology has provided beneficial technologies in pain management [14], cancer care [15], and cardiovascular risk reduction [16].

Despite growing recognition of the potential of co-creation, its systematic application in digital CR remains limited, and documentation regarding its impact on program development and implementation is scarce. Addressing this gap is crucial to inform best practices for designing patient-centered digital CR models that effectively translate into real-world settings. Therefore, the aim of this study was to provide a detailed description of the multidisciplinary co-creation process used to develop a person-centered, multimodal digital CR program that integrated the perspectives of stakeholders and end users throughout, with the rationale to optimize the acceptance and feasibility of the CR program.

## Methods

### Study Context

Despite improved treatment methods, 450,000 Norwegians live with chronic cardiovascular conditions, which not only affect their daily lives but also have implications for clinicians, health care systems, and society at large. The Patient-Reported Outcomes in Cardiology (PROCARD) research group focuses on patient-reported and clinical outcome measures to promote patient-centered care and treatment in cardiology. Through embedded collaboration with regional, national, and international hospitals, municipalities, and academic institutions, the consortium has initiated several large projects aimed at developing and researching equitable CR across the cardiovascular disease (CVD) population. Despite these efforts, many people diagnosed with CVDs are unable to reduce known risk factors for CVD. Therefore, PROCARD initiated the eCardiacRehab study embedded in clinical practice. The group has an interdisciplinary approach as the foundation for shared decision-making between patients and clinicians.

### Design

**Figure 1.**
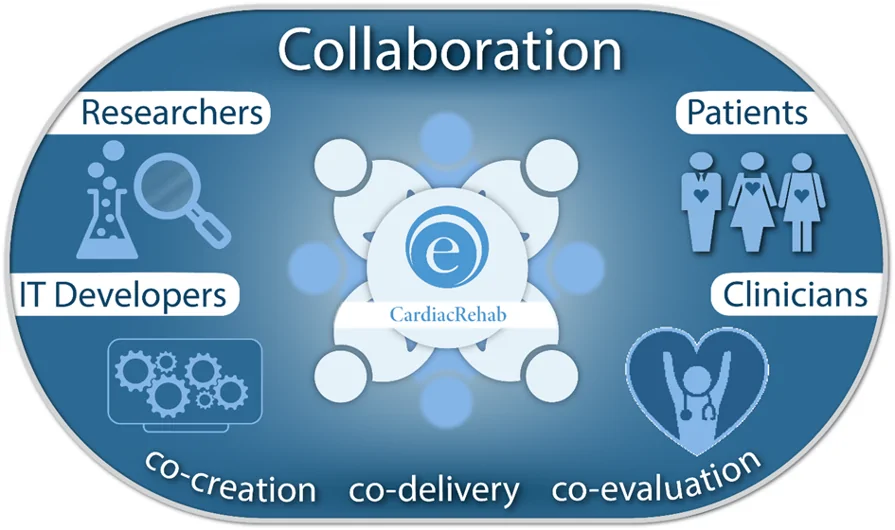
Central illustration of the co-creation process in the project.

**Figure 2.**
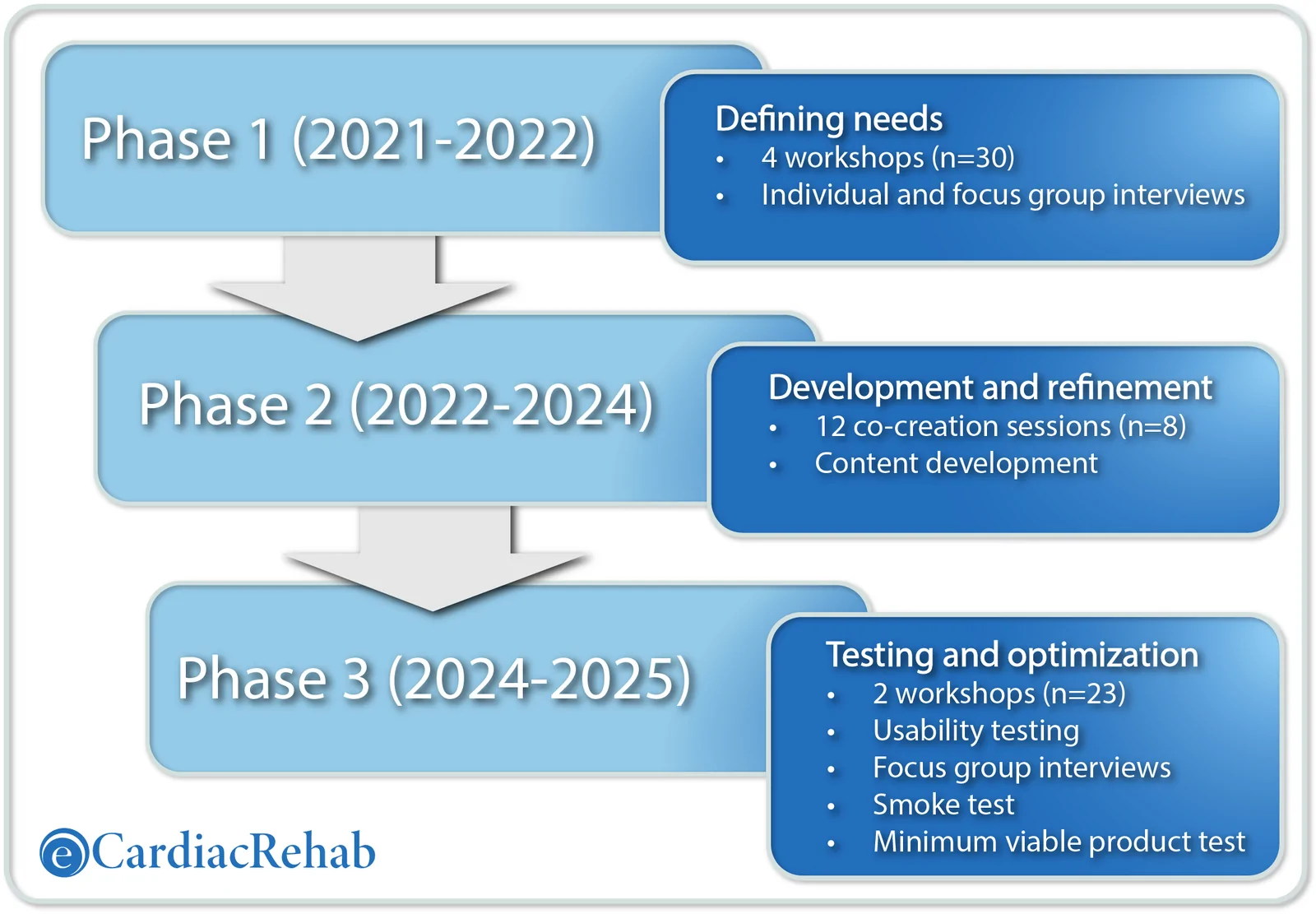
Overview of the phases in the project’s co-creation process.

Based on a user-centered design [17], the development process (Figure 1) of the digital CR program entailed an iterative 3-staged co-creation process (Figure 2) according to the Medical Research Council (MRC) framework [18]. The project used well-established multidisciplinary CR concepts and class 1A recommendations for secondary prevention of CVD from clinical guidelines [3] as an evidence-based foundation for the co-creation process. The development process was reported in accordance with the GUIDED (Guidance for Reporting Intervention Development Studies in Health Research) checklist (Checklist 1) [19].

The multidisciplinary project team consisted of researchers, clinicians, a software team of technology developers and designers, and user representatives, who helped ensure that content and materials were presented in an understandable manner. The project team was involved in all phases and met on a regular basis: monthly, weekly, and sometimes more often during the development process from January 2021 until the feasibility phase in January 2025.

### Recruitment

Health care and research participants were sampled purposively [20] to contribute their competence and experience to the co-creation process, reflecting the multidisciplinary nature of CR (eg, cardiology, nursing, physiotherapy, clinical nutrition, and pharmacology), cardiac and outpatient care (general practitioners [GPs], nurses, and cardiologists), eHealth development, and software and technology.

To be eligible as a user representative, the person had to be aged 18 years or older, have a diagnosis of CVD, and be Norwegian-speaking. A specific goal was to recruit a diverse group of user representatives across age groups and genders through the local branch of the National Association for Heart and Lung Diseases.

### Data Collection

The data material was compiled through a 3-phase, iterative process using several data sources (Table 1).

**Table 1.** Summary of data sources in the co-creation process of the project.

Phase	Type of data	Which stakeholder
1	Contextual inquiries 4 summaries from workshops 4 transcribed individual interviews	CONCARD^PCIa^ data (N=3417) [21-24] Multidisciplinary health care, academic, and IT professionals (n=30) General practitioners (n=4)
2	12 summaries from co-creation sessions	User representatives (n=8)
3	2 reports from usability testing 3 transcribed focus group interviews 1 report from smoke test 1 report from MVP^b^ test	User representatives (n=8, n=15) User representatives (n=8, n=7, n=8) Multidisciplinary health care professionals and ICT^c^ team

a CONCARDPCI: Continuity of Care After Percutaneous Coronary Intervention.

b MVP: minimum viable product.

c ICT: information and communication technology.

### Phase 1: Defining Needs

Phase 1 provided insights into end users’ needs through multiple methods: *contextual inquiry*, *stakeholder workshops,* and *individual interviews*, ensuring that the project was well anchored.

#### Contextual Inquiry

The contextual inquiry was based on substantial preparatory work from the CONCARD^PCI^ (Continuity of Care After Percutaneous Coronary Intervention) study, a large Norwegian longitudinal study of people with CAD (N=3417) [21], including a systematic review of modes of eHealth delivery in secondary prevention programs for people with CAD [22], and qualitative and quantitative studies [23,24]. These data provided information about the end users, their needs and requirements for acceptability, and the environment for which the program was designed. Overall, the CONCARD^PCI^ study pointed to the need for new digital CR interventions for people with CAD, including the assessment of health literacy and multimodal health education as important components of eHealth interventions [22,24]. Participants experienced unplanned journeys across care boundaries and did not receive adequate instruction and information on how to integrate health information. They also needed help to schedule clear follow-up appointments, and the study suggested the importance of early follow-up when the patients are motivated. Patients’ voices should be taken into greater account when working on follow-up care, pointing to the need for a co-creation approach [23,24].

#### Workshops With Stakeholders

Clinicians and researchers from collaborating institutions participated in workshops with the eCardiacRehab project team (n=30) facilitated by instructors trained in design thinking from InnoMed. InnoMed is a national competence network for needs-driven innovation in the health care sector, owned and managed by the 4 regional health authorities in Norway. Workshops were conducted digitally during the COVID-19 pandemic (2021‐2022). Service design methods using personas and patient journeys were used to facilitate user engagement based on existing research recommendations [25,26].

In the workshops, participants were divided into smaller groups of researchers and clinicians from various professional backgrounds. The information gathered in workshop 1 guided the subsequent 3 workshops, which focused specifically on the patient journey and the development process. Workshop 2 focused on recruitment, and workshops 3 and 4 focused on start-up and follow-up in the program. Notes from the workshop discussions were recorded, and materials from group tasks were collected.

**Figure 3.**
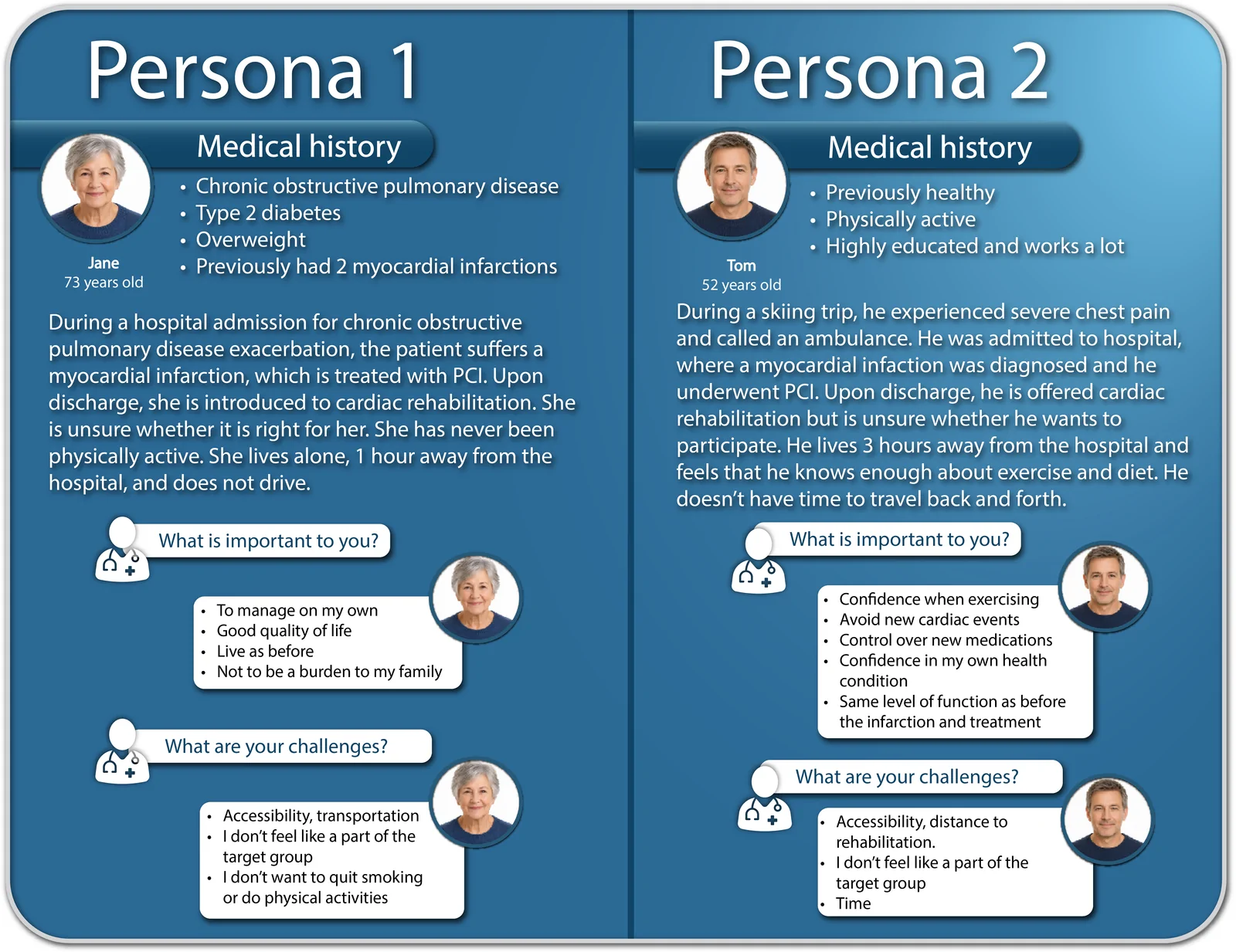
An example of the personas developed in the project. PCI: percutaneous coronary intervention.

Two fictional but representative patient profiles (*personas*; see Figure 3), and a patient journey map (Multimedia Appendix 1) visualizing typical patient pathways were developed [27]. The personas provided a shared understanding of the user profile, created contextual insight into the needs of end users, and helped to keep the focus on the users throughout the process. They represented diversity in CAD risk profiles, background, age, eHealth literacy level, health motivation and frustrations, relationships with clinicians, social support, and other influences on health behavior. The personas included background information (ie, stories to give each persona more depth), coping skills and everyday challenges, an overview of technology skills, and their needs and requirements regarding the eHealth program. The patient journey map described essential components of the rehabilitation pathway and highlighted important elements for digital CR and activities throughout the set period, including contact with clinicians and health care service levels (ie, hospitals and GPs).

Interviews with GPs (n=4) further informed user needs and how GPs could contribute to recruitment and follow-up care in a digital CR program. Data were analyzed using qualitative content analysis [28].

### Phase 2: Development and Refinement

Phase 2 included an iterative process consisting of 12 co-creation sessions and content development. Dedicated user representatives (n=8), aged 39 to 73 years (Table 2), participated in 12 co-creation sessions from July 2022 to December 2023. All participants lived in an urban area, and each co-creation session lasted approximately 2 hours, including 9 in-person meetings and 3 digital meetings. The purpose was to elicit ideas on design and content features and further explore users’ requirements for a digital CR program, with each session informing the next. First, a low-fidelity paper prototype, based on evidence-based topics, clinical guidelines, and logistics from local CR programs, was discussed. Throughout the process, the content was continuously shaped, adjusted, and adapted using an iterative approach based on user representatives’ feedback.

All co-creation sessions were facilitated by the second author (KJR) in collaboration with other team members (TMN, NH, TRP, and OKN) and documented in meeting minutes. The first author summarized the material and ensured that it provided essential input into the ongoing development process. The written material was analyzed using qualitative content analysis [28] and sorted into categories representing (1) content, (2) functionality, (3) design, and (4) barriers and facilitators to use (Table 3).

Furthermore, the development of the technology underwent several iterations depending on the topic, with the involvement of the information and communication technology (ICT) team and clinical experts to ensure the use of appropriate language in brief, readily understandable sentences suitable for computer screens. To evaluate the readability of the text, the Gunning Fog Index tool [29] was used. The tool is designed to estimate the years of formal education required to understand a piece of written text on the first reading.

**Table 2.** Characteristics of user representatives in co-creation sessions.

Sociodemographic characteristics	User representatives from co-creation sessions (n=8)	New group of user representatives (n=15)
Gender, n (%)		
Women	4 (50)	5 (33.3)
Men	4 (50)	10 (66.7)
Age (y)		
Range	39-73	51-85
Mean (SD)	58.3 (11.2)	69.3 (9.1)
Education, n (%)		
Primary school	—^a^	0 (0)
Upper secondary school	—	5 (33.3)
College or university	—	10 (66.7)

a Not available.

**Table 3.** Summarized results from 12 co-creation sessions in the project.

Categories	Principles	Comments
Content	Trustworthiness Personalization	Content developed from guideline recommendations for CR^a^ and aligned with place-based CR Information videos from experts in the field Qualified advice on risk factors, such as tips and menus for alternative diet choices, and exercise training programs on different levels of difficulty Information in common language and easy to understand Links to trustworthy external information Personal health data entry for self-monitoring page for medication, activity, diet, weight, blood pressure, nicotine use, and sleep diary Goal setting; important for motivation and evaluation Progression bar to see growth
Functionalities	Social support Features	Dialog by asynchronous message service Next-of-kin involvement Group-based activities Peer support Easy to navigate, menu function, and program have a logical setup Self-explaining graphs Videos and animations on all themes covered Knowledge quiz in modules to motivate
Design	Usability	Notifications, rewards, and reminders Pleasant color use and animations Universal design
Barriers and facilitators for use	Utility	Proper education in using the program Motivation in graphs, numbers, and progress bars Safety in physical test at start-up, contact persons, chat function, real-time exercise sessions Community, being part of a group Relatable user stories Nonstereotypical examples in program

a CR: cardiac rehabilitation.

### Phase 3: Testing and Optimization

The aim of phase 3 was to test and optimize the user interface and further develop the technical specifications and details of the program.

#### Usability Testing

The usability testing was conducted from March to April 2024 with user representatives from the co-creation sessions (n=8) and a new group of users (n=15) who were unfamiliar with the program.

The usability testing was conducted face-to-face by facilitators (KJR, TMN, TRP, and OKN) in a data laboratory, where all participants were granted identical computer systems and tools, including headphones (Multimedia Appendix 2). A think-aloud methodology [30] was used to actively engage the participants and elicit continuous feedback, with participants describing their actions and immediate thoughts for each step initiated by the facilitator, both orally and in writing on sticky notes. Observations were summarized following each test and sorted into information groups: (1) usability issues, (2) possible solutions, (3) reported issues, and (4) other input. This approach provided rapid, continuous, yet structured feedback for the development process.

The System Usability Scale (SUS) was used to assess acceptability. SUS measures usability and satisfaction on a scale from 1 (strongly disagree) to 5 (strongly agree). The sum score ranged from 0 to 100, with a cutoff at 68 indicating decent usability, 68 to 80.3 indicating good usability, and greater than 80.3 indicating excellent usability [31]. Data were analyzed descriptively using SPSS Statistics for Windows (version 29.0; IBM Corp).

#### Focus Group Interviews

After the usability testing, all 23 participants participated in focus group interviews (n=8, n=7, and n=8). The interviews were facilitated by the authors (OKN, TMN, KJR, and TRP) and had a general approach to evaluate the usability testing, the users’ experience of the process, and the program content. Data were transcribed verbatim and analyzed at the manifest level using qualitative content analysis [28].

#### Smoke Test and Minimal Viable Product Test

A smoke test and a minimal viable product (MVP) test were conducted by the research team and representatives from the IT developer, the Section for eHealth at the university hospital, and the Regional Hospital Trust IT (Helse Vest IKT). A smoke test is a preliminary test to ensure that the core functionalities of a software application are working correctly before more extensive testing is performed [32]. Key aspects include ensuring that the application launches successfully, that users can log in and out, that basic navigation within the application works, and that key features, such as search or data entry, are functioning. An MVP test was conducted to reduce risks and ensure a successful launch. It allows developers to gather user feedback early, iterate on the product based on real-world usage, and ultimately build a product that better meets users’ needs [33].

### Ethical Considerations

The study was reviewed and approved by the Regional Ethics Committee (REK 2015/57 and 459136) and registered at ClinicalTrials.gov (NCT06759805). User involvement was guided by the PRO-Ethics framework [34], a European Union Horizon initiative that emphasizes ethical, inclusive, and transparent participation in research and innovation. This framework supported the design of a participatory process that ensured all participants were treated with respect and had equal opportunities to contribute.

The participants were recruited through a regional patient organization, provided written informed consent, and were informed of the right to withdraw at any time. However, none of the participants withdrew from the study. The majority contributed to all co-creation sessions and participated in workshops, with only a few not participating due to an inconvenient schedule. All contributors were granted full anonymity during the process and in reporting of this study. Participants were involved at all levels of consultation and development, and their voices were given decision-making power in the different steps of the co-creation process, as described.

## Results

The 3-phase iterative co-creation process identified several steps of importance for stakeholders and end users, as summarized in Table 4 and detailed briefly in the following sections.

**Table 4.** Synthesis of insights from different data sources in the co-creation process.

Phase	Type of data	Insights	Synthesis
1	Contextual inquiries 4 summaries/minutes from workshops 4 transcribed individual interviews	Bottlenecks and opportunities in cardiac care, from the CONCARD^PCI^ study Insights (1) support to make lifestyle changes, understanding risk factors, and support for service development, (2) practical barriers and facilitators (ie, information, coordination, referral, and inclusion criteria) to recruit, (3) a suggestion for start-up of the program (ie, physical test and consultation with multiple clinicians covering risk factors and motivation), content in the digital CR^a^ program, and estimated costs and potential for funding	Start-up consultation Planned patient journey Goal setting Final consultation
2	12 summaries/reports from co-creation session	Insights into (1) content, (2) functionality, (3) design, and (4) barriers and facilitators to use	Tailored solution—start-up page with menu and personal health data entry for self-monitoring page Options for feedback—asynchronous message service Peer support—live workout sessions led by a physiotherapist and live medical yoga sessions Digital reminders—options for notifications and reminders Motivational features—9 interactive cardiac-specific learning modules
3	2 reports from usability testing 3 transcribed focus group interviews 1 report from smoke test 1 report from MVP^b^ test	Insights to (1) usability issues, (2) possible solutions, (3) reported issues, and (4) other input Insights highlighted the importance of motivation, through (1) personal contact throughout, and in the program, (2) to continue the behavioral change (eg, exercise and diet), linked to easy access, good information on the right level, motivational features, and a focus on “every step and activity is better than the sofa,” (3) important for the participants to feel safe, that they were assessed by qualified clinicians prior to startup of the program and that they were not on their own. The reach of the program and program fit for different participant groups (ie, women and older adults) were emphasized.	Inclusive of phase 2 synthesis

a CR: cardiac rehabilitation.

b MVP: minimal viable product.

### Phase 1: Defining Needs

#### Workshops With Stakeholders

The initial workshops with stakeholders (n=30) contributed to anchoring the project in the different system levels of health care service. From the developed personas and patient journey, participants identified several ways that a digital CR program could meet users’ needs, including support for making lifestyle changes, understanding CAD risk factors, and support for service development. Core elements, including practical barriers and facilitators (ie, information, coordination, referral, and inclusion criteria), to recruitment were discussed. The workshops provided feedback on the start-up of the program (ie, physical testing and consultation with multiple clinicians covering risk factors and motivation), the content of the digital CR program, and suggestions regarding the estimated costs and financial opportunities in the health care system.

#### Interviews With GPs

GPs expressed that a digital solution would be suitable for reaching more patients and offering a comprehensive service that could uphold patients’ daily lives, as they could continue working during participation in the program. Furthermore, GPs saw themselves as motivators for healthy lifestyle changes through a digital CR program, actively referring people to CR, and viewed the digital CR program as a learning tool to better enhance patients’ resources.

### Phase 2: Development and Refinement

**Figure 4.**
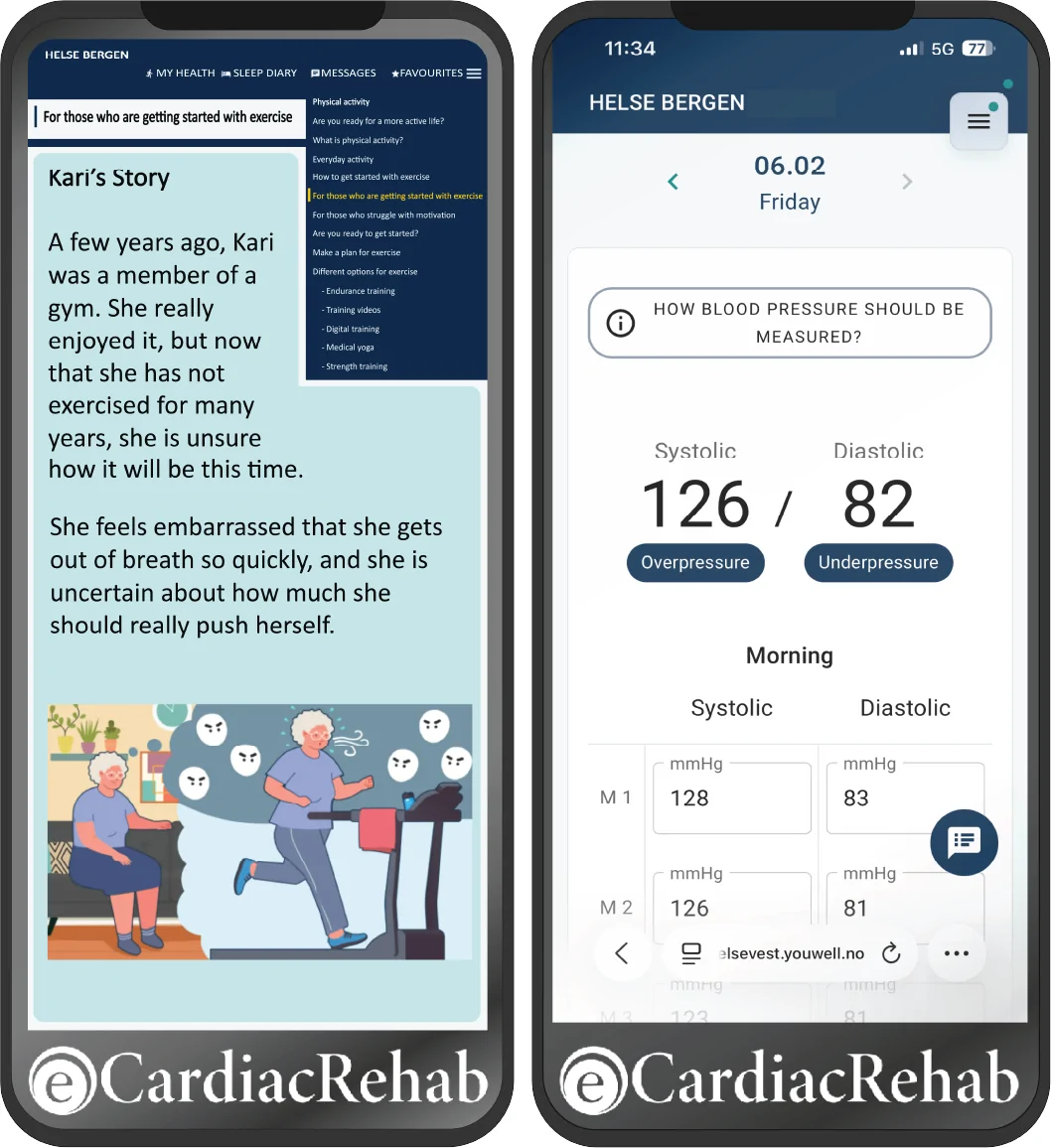
Example of cardiac rehabilitation trajectory and data entry for self-monitoring module developed in the process.

The 12 co-creation sessions contributed to the development of the prototype from low to high fidelity. The target audience representation was adequate, as the participants included both women and men within the target age range who had CVD, and all used a mobile phone and/or computer in daily life. Prototypes of increasing fidelity were developed, with testing of the content, animations, videos, language, navigation, and labels, including appealing features such as the personal health data entry for self-monitoring (Figure 4).

Throughout the co-creation sessions, a range of viewpoints and preferences regarding the use of a digital CR program were revealed. The role of technology in day-to-day life varied in the group. While most users foresaw great benefits from a digital CR solution, some also highlighted uncertainty about its benefits. Comments made by the user group illustrate the link between categories and end-product features representing (1) content, (2) functionality, (3) design, and (4) barriers and facilitators to use (Table 3). At the start of the co-creation sessions, users listed their preferred attributes for the digital program, highlighting the following: a physical consultation at the start of the program, a chat function during the rehabilitation period, counseling on diet, the involvement of next-of-kin, goal-setting, and progression measures. During the co-creation sessions, users had access to high-fidelity prototypes and specific content, such as modules, animations, and videos. An example of feedback was the setup of the modules, where the module on psychosocial health was deemed so important that it was prioritized as the third component in the sequence. Another example was the suggestion to develop a module on female heart health and a resource library to gather trustworthy information. Although the number of participants in the group was small, a saturation effect in terms of the feedback and input from participants was reached.

Building on the feedback and discussions within the project team, a prototype was developed that included a start page with a menu and 9 cardiac-specific interactive learning modules in 5 main components: (1) physical activity and exercise, (2) healthy lifestyle education, (3) psychosocial health, (4) self-monitoring and self-management, and (5) medication adherence. Furthermore, a registration module for health data entry for self-monitoring (medications, weight, blood pressure, blood sugar, activity, diet, sleep, mental health, and nicotine status) was designed (Figure 4). The program also includes real-time guided workout sessions led by physiotherapists, real-time medical yoga sessions, and educational videos in all modules. An asynchronous messaging service and scheduled digital consultations ensure communication with the multidisciplinary CR team throughout the program. Moreover, 2 user stories were developed to illustrate different CR trajectories (Figure 4), including variations in acute care pathways across the program’s modules. These user stories were included in the program to provide patients with relatable scenarios, compensating for the lack of peer interaction typically present in center-based CR. The readability screening revealed a suitable Gunning Fog Index, with optimal readability levels identified as falling within the range of 4 to 13. This indicates that the text is comprehensible to readers with 9 or fewer years of formal education. The overall SUS score was 86.2 (SD 12.2), indicating excellent usability.

### Phase 3: Testing and Optimization

#### Interviews With Focus Group

The focus group interviews following usability testing provided rich conversations and further informed the program development. Participants highlighted the importance of motivation, both through personal contact throughout the program and within the program. Motivation to continue the behavioral change (ie, exercise and diet) was linked to easy access, information at the right level, motivational features, and a focus on “every step counts, and activity is better than the sofa.” To be able to participate in a digital CR program, it was important for the participants to feel safe; they wanted reassurance that an elevated heart rate was not worrisome, that they could exercise safely at their own pace, that they were assessed by qualified clinicians prior to the start of the program, and that they were not on their own. The reach of diverse groups with a program that fit all users was emphasized.

The second and third focus group interviews were conducted with participants (n=7 and n=8) unfamiliar with the program. Several had previously participated in center-based CR and stated that if the digital solution had been available to them at that time, they would have preferred digital CR over center-based CR. They expressed a need for trustworthy and validated content and reported that searching the internet often was a suboptimal solution; hence, this program filled an information gap. The program was considered user-friendly, with a good overview and easy to access and use. One participant said:

*I also think it’s fun to be able to help develop something that others will need later, and something that I was looking for myself during the first months before I started CR, because there was little information then. I was Googling. It’s nice to have everything in one place in a programme like this*.

[P7]

Some had concerns regarding the lack of fellowship with peers during the CR period and expressed that this would be absent in a digital solution, in both educational and training sessions. Still, there were those who preferred home-based group exercise rather than having to travel a long distance. They also appreciated the granular information level and asked for digital access to the program for next-of-kin. They saw themselves as curious and interested in digital solutions.

#### Smoke Test and MVP Test

In total, 33 defects were detected during the smoke test. Of these, 4 were categorized as “B—Major” and 29 as “C—Minor” according to the application life cycle management (ALM) system. The major defects included a lack of an alert when participants repeatedly registered elevated blood pressure, the option to register medication consumption for a previous period, malfunctioning links to external web pages, and the inability of clinicians to tailor the program according to patients’ needs (ie, to remove the nicotine module if the patient was a nonsmoker). Minor defects were related to data entry for self-management, visualization of data entry, missing text in educational modules, typographical errors, and registrations not being shown on the clinician’s dashboard.

Furthermore, at the time of testing, the program was delivered as a responsive web application rather than a native application. Therefore, display and navigation flaws were detected when the content was viewed on smaller mobile phone screens. In order to avoid these challenges, eCardiacRehab was made available both as a web and mobile application (hybrid app). The mobile application can be used on both Android and iOS platforms. Furthermore, the possibility of using biometrics when logging in was established.

In total, 108 defects were detected during the MVP test. Of these, 6 were categorized as “B—Major,” 99 as “C—Minor,” and 3 as “D—Observation” according to the ALM system. The major defects were related to a lack of contrast in the visualizations of patients’ health data entry, and thereby not adhering to the legal requirements for universal design of ICT in Norway.

#### Privacy by Design, Software Flow, and Technical Architecture

The CR program is developed in accordance with the principles of Privacy by Design. This entails that the system is designed and maintained with data protection as a core requirement. Measures include security testing, secure coding practices, quality standards for design and implementation, and robust procedures for the operation and governance of the information system. Regular penetration testing is performed to identify and mitigate potential vulnerabilities.

The design and development were in accordance with the European General Data Protection Regulation of 2018 [35]. Stringent access controls and high-security requirements (level 4) for authentication were implemented, in addition to safeguards ensuring adherence to fundamental data protection principles. Access to personal data is strictly limited to the patient’s clinician, the system administrator, and designated superusers within the hospital. The digital tool operates on a one-to-one basis, meaning that each patient is linked to their respective clinician. Comprehensive logging functionality ensures full traceability. The tool also provides an overview of assigned access rights. A formal log review procedure to be carried out by the system administrator has been established.

The cloud solution Microsoft Azure is used as a subcontractor, with a data center located in Norway, which is risk-assessed by the regional hospital trust’s ICT department. The solution is hosted in Azure using platform as a service (PaaS). App services are used for APIs and websites, and Microsoft SQL Database is used for databases. All data transmission is encrypted over HTTPS and complies with the requirements of the Norwegian National Security Authority and Section 2.2 of the NORMEN (Norwegian Code of Conduct for Information Security and Data Protection in the Health Care Sector) guidelines.

The database (MS SQL) is encrypted with transparent data encryption. This provides “encryption at rest,” which prevents data from being read in the event of a data breach or a similar incident that provides direct disk access to the database files.

A risk assessment analysis of the platform (ROS-ID 965) and the use of activity trackers (ROS-ID 25043) was performed. Furthermore, a data protection impact assessment was conducted (2025/2926‐4).

## Discussion

### Principal Findings

In this study, we describe the iterative co-creation process and design of eCardiacRehab, a multimodal digital CR program. To our knowledge, this is one of the few studies that combine thorough stakeholder and user involvement and research their preferences. Moreover, this approach combined stakeholder and user perspectives with evidence-based knowledge to develop a digital home-based CR program that covers all guideline-directed components of CR.

The development process combined evidence-based and user-centered approaches, a recommended but underused approach to the development of eHealth solutions [36]. In this study, we incorporated all guideline-recommended modalities of CR. Although some initiatives, such as CoroPrevention [37] and INTERCEPT [38], have recently taken the multimodal approach forward, prior digital interventions have focused primarily on physical activity or exercise training and have lacked the full spectrum of core components of CR, such as lipid and diabetes management, nutrition, psychosocial support, and smoking cessation [39]. Given the importance of contextual factors in shaping intervention uptake and effectiveness, interventions should be adapted to local, regional, and national circumstances. Co-designing interventions within users’ own context can help ensure that the intervention is aligned with user needs, priorities, and implementation conditions [10,36]. Our iterative co-creation process identified 5 themes of importance for stakeholders in meeting the end users’ needs. These themes included a tailored solution, options for feedback, peer support, digital reminders, and motivational features. Areas for improvement regarding the 5 themes were identified through contextual inquiries, stakeholder workshops, and iterative content development based on evidence-based strategies for CR. In general, ways to optimize the co-creation process from a user perspective are missing [10], and our results suggest avenues for shaping the process through the use of moderators, guidance, and continuous engagement.

### Patient Involvement

This study highlights the importance of thorough patient involvement in co-creation sessions over an extended period. Such engagement is not merely beneficial but essential for understanding and integrating the needs and contexts of intended users [40], emphasizing how critical it is to prioritize user perspectives in the development of effective eHealth solutions [41,42]. A recent systematic review [41] identified usability and user-friendliness as prominent technology-related factors for health care professionals’ adoption of digital health interventions. This underscores the value of co-design methodologies in developing interventions that are both acceptable and implementable in clinical practice. Continuous patient involvement is essential not only for maintaining engagement but also for building trust and ensuring that participants feel safe and valued throughout the process [42]. It enabled users to address the core needs for a tailored solution, including options for feedback, peer support, digital reminders, and motivational features. Adopting a tailored approach is crucial for enhancing digital CR programs [43]. Engaging patients throughout the co-creation trajectory enabled the collection of essential feedback that informed the design decisions. The MRC framework [18] emphasizes that meaningful engagement of patients can maximize the potential of novel interventions that are likely to positively affect health. Empirical evidence substantiates that active user participation in development enhances the likelihood of successful adoption and seamless integration into users’ daily lives [11,44]. Furthermore, user involvement serves to diminish barriers to technology adoption, fostering a sense of ownership and agency among users, both of which are crucial determinants of the sustainability and effectiveness of a digital CR program [45].

### Principal Methodology

Patient and public involvement is essential when developing eHealth interventions [18]. We initiated the co-creation process involving stakeholders in workshops using personas, which are fictional representations of characteristic real users [27], to better target the end users’ needs. The deployment of personas can function as an instrumental mechanism for acquiring a nuanced understanding of the user experience, fostering empathy, and elucidating user needs [46]. We constructed robust personas that helped focus the stakeholder workshops on the actual needs and goals of patients. A co-creation process is time-consuming due to the necessity for multiple feedback cycles, and it requires significant resources, both financial and human [11]. The use of personas may contribute to less extensive processes and contribute to the enhancement of self-management behaviors in digital CR [47]. This program was developed to engage a broad range of users, promote diversity, and address inequities in eHealth solutions [48]. Personas may elucidate how diverse patient demographics—such as women, older adults, or individuals with multiple comorbidities (persona 1)—exhibit heterogeneous requirements that necessitate tailored CR programs. Personas may also help narrow the design options, thereby enhancing the effectiveness of co-creation processes [36]. Using personas may also facilitate deeper user engagement, thereby advancing the overall user experience when developing a digital CR program [27].

### Strengths, Limitations, and Future Directions

The active user participation throughout the design process, with a diverse and representative group, is a strength that can lead to increased adoption and advocacy of the program. The same principal investigator (PI) and team followed the process and supervised all development phases, ensuring continuity in all aspects. The PRO-Ethics framework [34] was essential in enabling meaningful engagement and ensuring that participants’ perspectives were genuinely integrated into the development process.

However, this process was not without challenges. Despite strong user involvement, users may lack the technical expertise to provide insightful feedback on all aspects of the design. Therefore, while co-creation holds substantial promise for driving innovation and fostering user-centered design, it necessitates careful management to balance its benefits against inherent challenges.

One key issue is that eHealth approaches are not finalized after a pilot or main trial, but need a strategy for co-creation and revision across the full life cycle. As co-creation processes continue to evolve, there are several future directions to enhance their effectiveness and impact on health service development. One promising avenue is the integration of advanced technologies, such as AI and machine learning, into co-creation platforms in health care [49]. These technologies have the potential to analyze user data more effectively, providing objective insights that can help tailor the co-creation experience by identifying patterns beyond and across individual user preferences and behaviors.

### Conclusions

This study offers insights into how to take a user-centered approach to the design and development of an evidence-based digital CR program to secure a tailored solution. The study involved stakeholders and users in all the phases of the iterative process, pointing out important steps for developing useful and meaningful interventions for patients using stakeholder workshops and co-creation sessions. In addition, to inform the development process, this study provides a practical example for future studies. Co-creation sessions with patient engagement over an extended period and the use of personas are recommended. To test usability, feasibility, and acceptability, as well as the potential efficacy of the program, further research is needed. A feasibility study is currently underway to optimize eCardiacRehab in preparation for a full-scale randomized controlled trial.

## Supplementary material

10.2196/93398 Multimedia Appendix 1 Example of a journey map developed in the eCardiacRehab.

10.2196/93398 Multimedia Appendix 2 Image 1: picture from usability testing laboratory.

10.2196/93398 Checklist 1 GUIDED checklist.

## Abbreviations

ALM: application life cycle management
CAD: coronary artery disease
CONCARD^PCI^: continuity of care after percutaneous coronary intervention
CR: cardiac rehabilitation
CVD: cardiovascular disease
GP: general practitioner
GUIDED: Guidance for Reporting Intervention Development Studies in Health Research
ICT: information and communication technology
MRC: Medical Research Council
MVP: minimal viable product
NORMEN: Norwegian Code of Conduct for Information Security and Data Protection in the Health Care Sector
PaaS: platform as a service
PCI: percutaneous coronary intervention
PI: principal investigator
PROCARD: Patient-Reported Outcomes in Cardiology
SUS: System Usability Scale
